# miR-709 exerts an angiogenic effect through a FGF2 upregulation induced by a GSK3B downregulation

**DOI:** 10.1038/s41598-024-62340-4

**Published:** 2024-05-18

**Authors:** Koji Ueno, Hiroshi Kurazumi, Ryo Suzuki, Masashi Yanagihara, Takahiro Mizoguchi, Takasuke Harada, Noriyasu Morikage, Kimikazu Hamano

**Affiliations:** 1 Department of Surgery and Clinical Science, Graduate School of Medicine, Yamaguchi University, Minami-Kogushi 1-1-1, Ube, Yamaguchi 755-8505 Japan; 2 Division of Advanced Cell Therapy, Research Institute for Cell Design Medical Science, Yamaguchi University, Ube, Yamaguchi Japan

**Keywords:** miRNA, Angiogenesis, miR-709, GSK3B, FGF2, Extracellular vesicles, Regenerative medicine, miRNAs

## Abstract

The aim of this study was to identify angiogenic microRNAs (miRNAs) that could be used in the treatment of hindlimb ischemic tissues. miRNAs contained in extracellular vesicles (EVs) deriving from the plasma were analyzed in C57BL/6 mice, which have ischemia tolerance, and in BALB/c mice without ischemia tolerance as part of a hindlimb ischemia model; as a result 43 angiogenic miRNA candidates were identified. An aortic ring assay was employed by using femoral arteries isolated from BALC/c mice and EVs containing miRNA; as a result, the angiogenic miRNA candidates were limited to 14. The blood flow recovery was assessed after injecting EVs containing miRNA into BALB/c mice with hindlimb ischemia, and miR-709 was identified as a promising angiogenic miRNA. miR-709-encapsulating EVs were found to increase the expression levels of the fibroblast growth factor 2 (FGF2) mRNA in the thigh tissues of hindlimb ischemia model BALB/c mice. miR-709 was also found to bind to the 3′UTR of glycogen synthase kinase 3 beta (GSK3B) in three places. GSK3B-knockdown human artery-derived endothelial cells were found to express high levels of FGF2, and were characterized by increased cell proliferation. These findings indicate that miR-709 induces an upregulation of FGF2 through the downregulation of GSK3B.

## Introduction

It has been reported that 15–20% of the people being over 70 years old suffer from peripheral arterial disease (PAD)^1^, and it is estimated that 200 million patients suffer from PAD in the world^2^. Approximately 1–3% of the PAD patients that are over 50 years old are critical limb ischemia (CLI) patients^1,3^. The new CLI patients every year are estimated at 500–1,000 people per million of the population in Europe or North America.1 Most CLI patients receive an endovascular treatment through the use of stent grafts and bypass surgery as a first choice of treatment in order to achieve the required revascularization^4^. However, 20–30% of the CLI patients cannot undergo a revascularization surgery, as they do not meet the eligibility criteria for the operation required^5^. As a result, one year after receiving a primary treatment, 45–50% of the CLI patients have both limbs without any amputation, 25–30% of the CLI patients have had to undergo an amputation, and 25% of CLI patients have died^1,3,4,6^. Therefore innovative angiogenesis-inducing therapies are required in order to reduce both the number of patients undergoing an amputation and the CLI-associated mortality rate.

Cell therapy has been shown to improve the blood flow in the ischemic limbs of CLI patients with no treatment options^5–7.^ We have reported the effects of the transplantation of autologous bone marrow cells and peripheral blood mononuclear cells in CLI patients^7,8.^ It is believed that the mechanism of neovascularization as a result of a cell transplantation is based on the paracrine effects mediated by the growth factors, cytokines, chemokines, and extracellular vesicles (EVs) secreted from the transplanted cells; these effects seem to promote cell growth in the recipients, and facilitate the regeneration of their tissues^7,9.^

Cells can release EVs of various sizes. Exosomes are an example of EVs that are defined as vesicles of a diameter lower than 150 nm^10^. The MISEV2018 statement has advocated in favor of the use of a nomenclature based on the size of the EVs^11^. As a result, EVs have recently been classified into small EVs (sEVs), medium EVs (mEVs), and large EVs (lEVs), defined by sizes < 100 nm, < 200 nm, and > 200 nm, respectively. Exosomes secreted from human CD34 + cells have been shown to exert the same angiogenic potential as the human CD34 + cells in a mouse hindlimb ischemia model^9^. In the latter case, the angiogenic potential was attributed to the miR-126-3p contained in these exosomes.

microRNAs (miRNAs) are non-coding RNAs containing approximately 22 nucleotides. miRNAs bind to the 3′UTR (untranslated region) of the target mRNAs, and inhibit translation^12^. The human and mouse miRNA sequences registered at the miRBase (https://www.mirbase.org/) are 1,917 and 1,234, respectively. We have previously reported that the miRNA-762 and the miRNA-3072-5p target the vascular endothelial growth factor (VEGF), which is one of the most important factors associated with angiogenesis^13^. It has been reported that miR-93 is related to angiogenesis, as (i) it has been found to be upregulated in the ischemic muscular tissue of C57BL/6 mice, and (ii) it has been associated with angiogenesis based on a comparative analysis of mice with different responses to the experimental simulation of hindlimb ischemia^14^. It has also been shown that the inhibition of the expression of miRNA-329, miRNA-487b, miRNA-494, and miRNA-495 encoded on the chromosome 14q32 in the ischemic muscular tissue of a C57BL/6 hindlimb ischemia model, can increase neovascularization^15^.

In this study, we identified suitable EV-contained miRNAs through a three-step screening process involving: (i) a comparative analysis of the EV-encapsulated miRNAs between C57BL/6 and BALB/c mice that exhibit different responses to the experimental simulation of hindlimb ischemia, (ii) an in vitro aortic ring assay, and (iii) the assessment of the blood flow recovery after injecting EV-contained miRNAs into BALB/c mice with hindlimb ischemia. We also undertook molecular analysis and further in vitro experiments in order to explore the mechanisms underlying the angiogenic effect of an identified angiogenic miRNA: miR-709.

## Results

### miR-709 was selected as an angiogenic miRNA by screening methods

**Figure 1:**
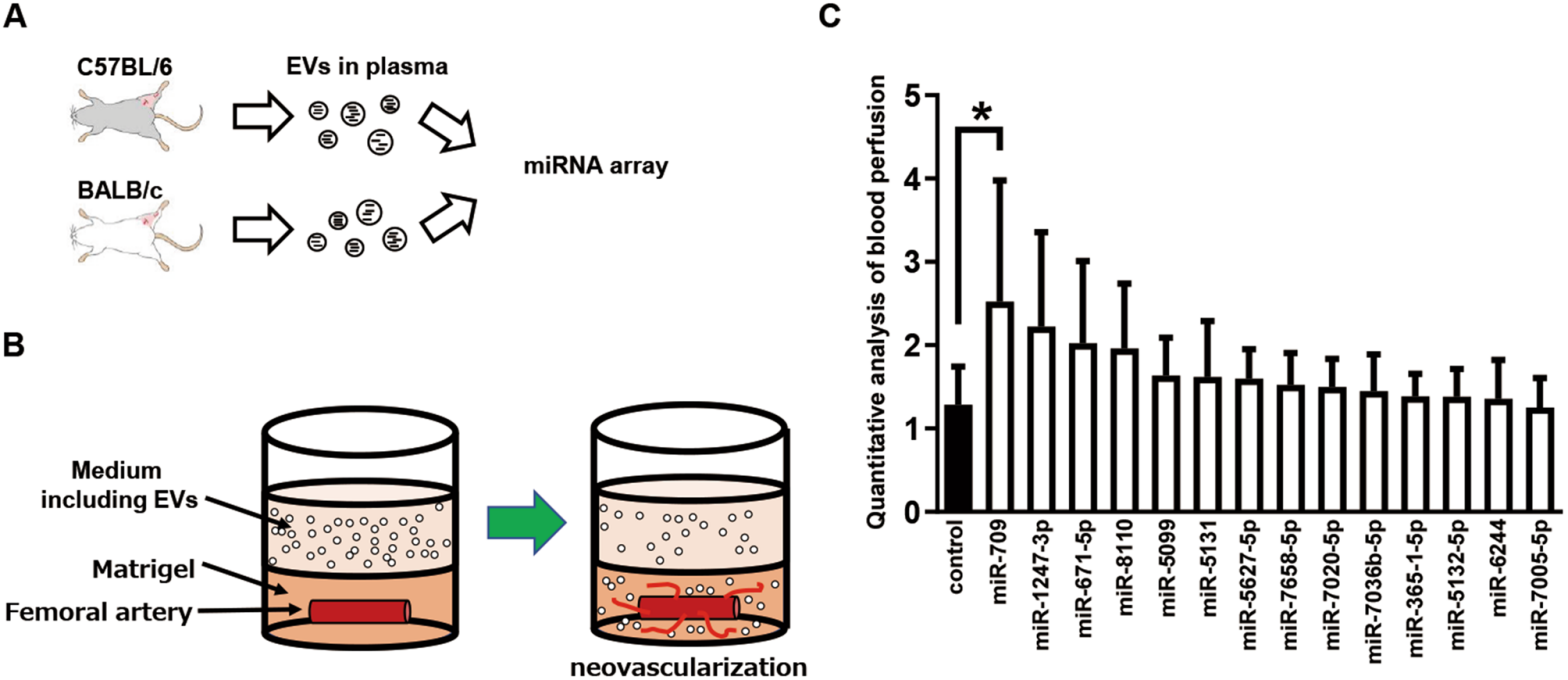
miR-709 was selected as an angiogenic miRNA by screening methods. (**A)** The first screening was performed by focusing on the characteristics of different angiogenesis levels exhibited by different mouse strains. miRNAs were extracted from EVs that were isolated from the mouse plasma at 24 h after the ligation of the left femoral artery and vein. miRNA-pooled samples were analyzed by a miRNA array. (**B)** Aortic ring assay was carried out as part of the second screening. Femoral arteries deriving from BALB/c mice were embedded in Matrigel and were co-cultured with miRNA-including EVs. This experiment was carried out three times, independently. In case a neovascularization was observed even in one out of the three repeats, the respective miRNAs were classified as angiogenic miRNA candidates. (**C)** The blood flow in the hindlimb ischemic model BALB/c mice was measured after injecting miRNA-containing EVs; this was the third screening. Blood perfusion at POD15 was normalized by POD1. Each group consisted of six mice (n = 6; Dunnett’s multiple comparisons test). Bars represent means with standard deviations (SDs).

During the first screening we compared the expression levels of miRNAs in EVs isolated from plasma between hindlimb ischemia model C57BL/6 mice exhibiting a blood flow recovery after a ligation of their femoral vessels and hindlimb ischemia model BALB/c mice not exhibiting such a blood flow recovery (Fig. 1A; Supplementary Data). miRNAs No1-No31 were only detected in the C57BL/6 mouse EVs at 24 h after ligating the left femoral artery, but were not detected in the BALB/c mouse EVs. The expression levels of miRNAs No32-No43 were more than 20-fold higher in the C57BL/6 mouse EVs as compared to the BALB/c mouse EVs (Supplementary Table 1). The 43 miRNAs identified by the first screening are shown in Supplementary Table 1.

In order to observe their angiogenic potential (if any), an aortic ring assay was performed by using the EVs that were secreted from 293 T cells transfected with plasmids coding different miRNA sequences (Fig. 1B). Neovascularization was observed in 14 EVs out of the 44 EVs assessed. The 14 miRNAs with angiogenic potential that were identified by the second screening are shown in Supplementary Table 1.

EVs were injected into the femoral muscles of the hindlimb ischemia model mice, and their blood flow was analyzed (Fig. 1C). There was a statistically significant difference caused by the EVs that were secreted from 293 T cells transfected with plasmids encoding miR-709 (as compared to control that were secreted from 293 T cells transfected with a mock plasmid).

### Characterization of the EVs released by 293 T cells transfected with miRNA mimics

**Figure 2:**
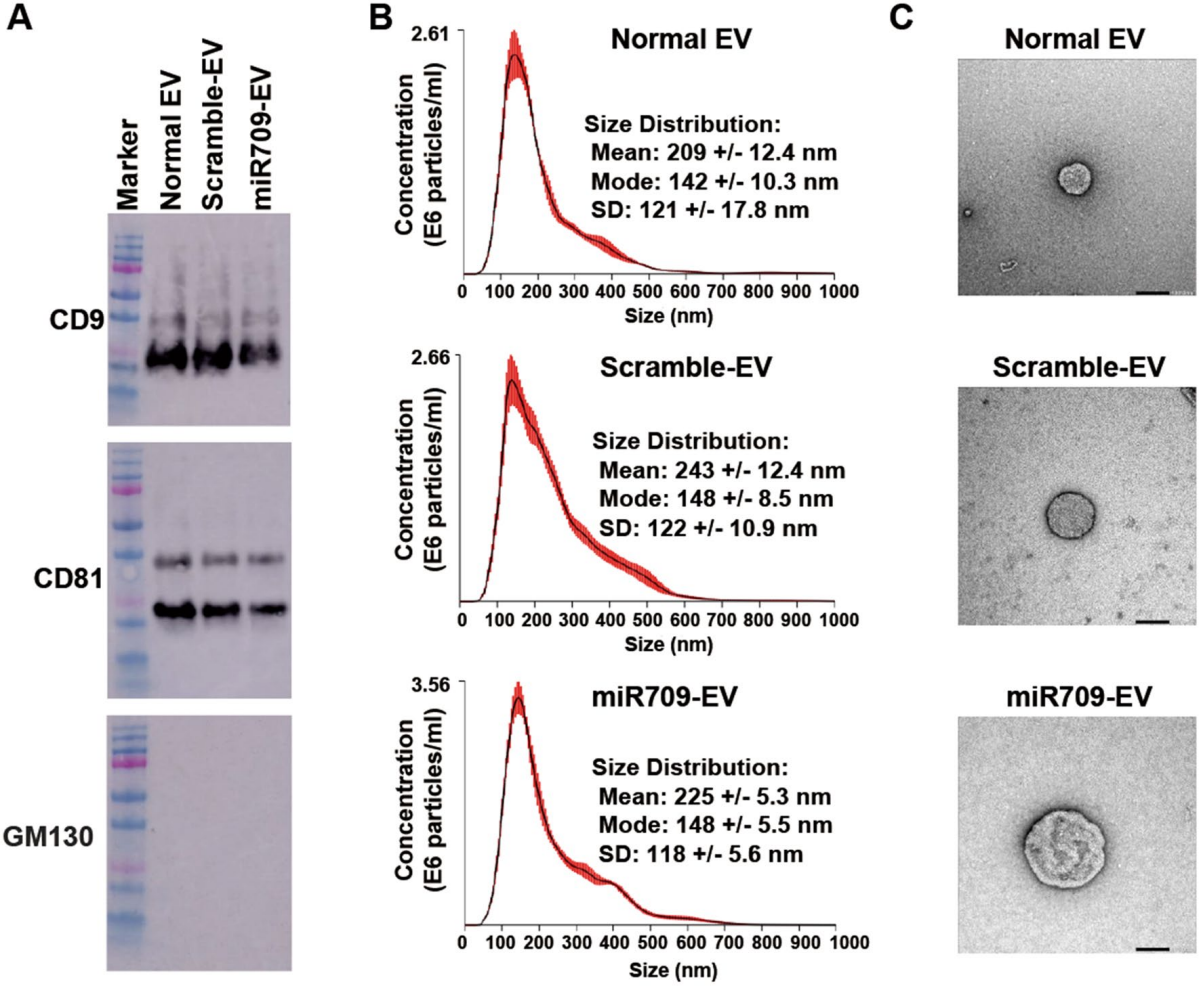
Characterization of the EVs released by 293 T cells transfected with miRNA mimics. (**A)** Western blotting was performed so as to examine markers; CD9 and CD81 acted as positive controls for EVs, while GM130 was a negative control for EVs. Figures were full-length blots with colorimetric marker and did not undergo any image processing. (**B)** The size of the EVs was analyzed by nanoparticle tracking analysis. The same samples were measured five times. Red error bars indicate ± 1 standard error of the mean. (**C)s** EVs were observed through a transmission electron microscope. The black bar represents 100 nm.

In order to analyze the characteristics of the miRNA-containing EVs, three types of experiments were carried out (Fig. 2). EVs bear CD9 and CD81 (markers of EVs), but no GM130 (a Golgi apparatus marker) (Fig. 2A). The mean size of the EVs was 209 ± 12.4, 243 ± 12.4, and 225 ± 5.3 nm in normal, scramble, and miR-709-containing EVs (Fig. 2B). The morphology of the EVs was round (Fig. 2C).

### miR-709 upregulated the FGF2 expression both in vitro and in vivo

**Figure 3:**
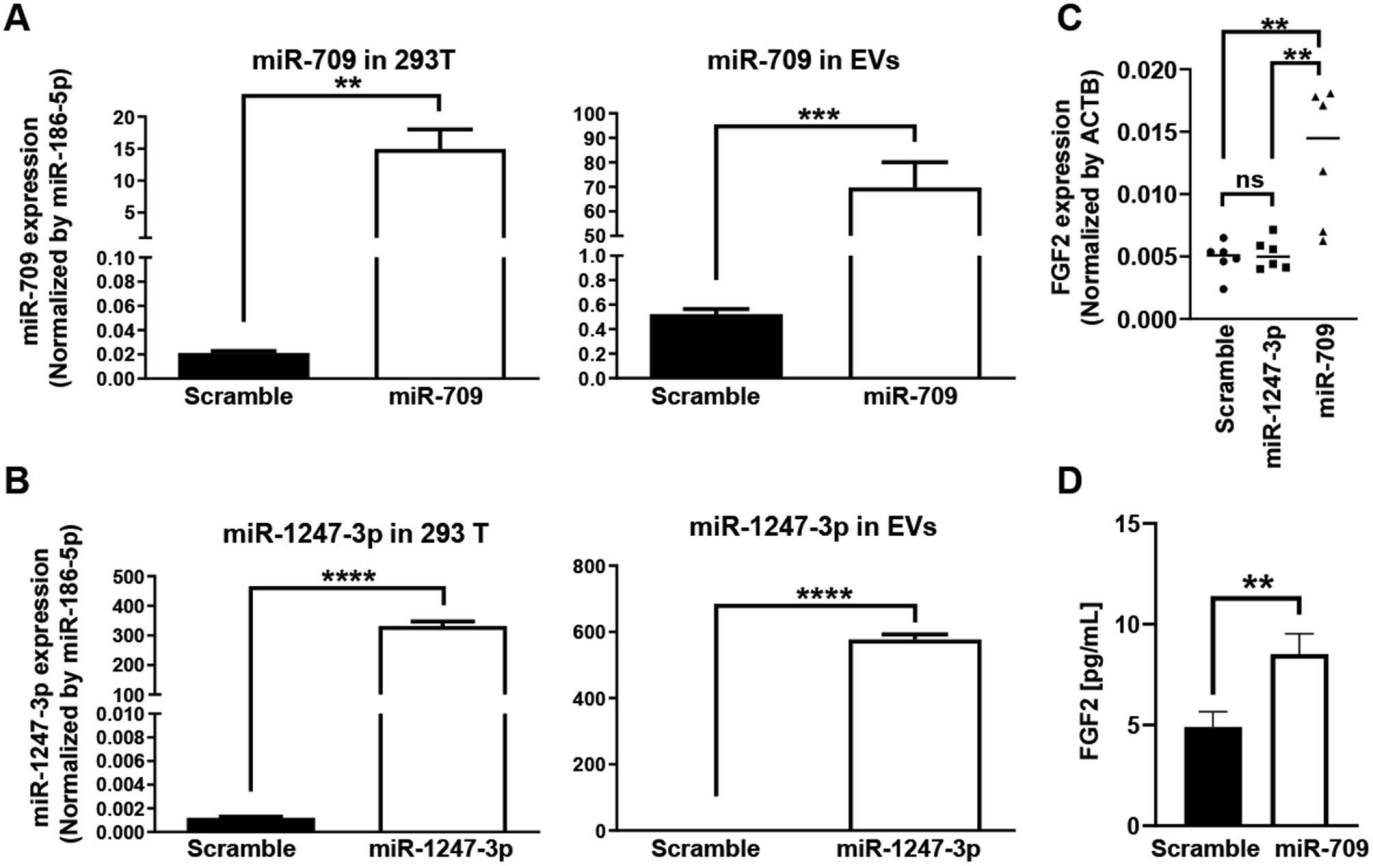
miR-709 upregulated the FGF2 expression both in vitro and in vivo. (**A**, **B)** miRNA mimics were transfected into 293 T cells and the expression levels of the miRNA mimics were analyzed in 293 T cells (left figures) and in the EVs that were released from the 293 T cells (right figures) by using qPCR. miR-709 and miR-1247-3p were normalized by miR-186-5p (n = 3; unpaired t-test). A presents the miR-709 data, and B presents the miR-1247-3p data. (**C)** The expression levels of FGF2 were analyzed in the thigh muscles after an injection of miRNA-encapsulating EVs. miRNA-encapsulating EVs were injected into the left thigh muscle at 4 days after the ligation of the left femoral artery, and the mRNA expression levels were analyzed at 2 days after the injection of the EVs. FGF2 was normalized by ACTB. Each group consisted of six mice (n = 6; Tukey’s multiple comparisons test). Lines represent the median. (**D)** The FGF2 concentration was measured by ELISA in HAoECs transfected with miRNA mimics. HAoECs were transfected with a scramble mimic or a miR-709 mimic. Protein was extracted at 3 days after the transfection. The FGF2 concentration was measured by ELISA (n = 3; unpaired t-test). Bars represent means with SDs.

In order to examine the inclusion amount of miRNA in the EVs secreted from miRNA-transfected 293 T cells, a qPCR was performed. The inclusion amount of miR-709 was 133-fold higher in EVs secreted from miR-709-transfected 293 T cells than in EVs secreted from scramble-transfected 293 T cells (Fig. 3A). The inclusion amount of miR-1247-3p was 577-fold higher in EVs secreted from miR-1247-3p-transfected 293 T cells than in EVs secreted from scramble-transfected 293 T cells (Fig. 3B). In an attempt to evaluate the angiogenic mechanism associated with miR-709, the expression levels of the FGF2 mRNA were assessed in the femoral muscles of hindlimb ischemia model mice after the injection of miR-709-containing EVs. Although the expression levels of the FGF2 mRNA in miR-1247-3p-EVs were the same as those in scramble-EVs, the expression levels of FGF2 mRNA in miR-709-EVs were found to be significantly higher than those of the scramble-EVs and of the miR-1247-3p-EVs at 2 days after the EVs’ injection (Fig. 3C). In order to confirm whether the miR-709 was related to the upregulation of FGF2, a miR-709 mimic was transfected to HAoECs and the FGF2 concentration in HAoECs was analyzed by using ELISA. The FGF2 concentration increased by 1.7-fold in the miR-709-transfected HAoECs (as compared to the scramble-transfected HAoECs) at 3 days after the transfection (Fig. 3D).

### miRNA-709-encapsulating EVs upregulated the expression levels of FGF2 and BMX in HAoECs

**Figure 4:**
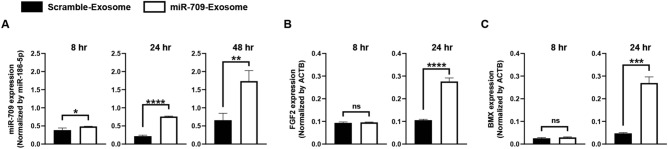
miRNA-709-encapsulating EVs upregulated the expression levels of FGF2 and BMX in HAoECs. (**A)** miR-709 expression levels were analyzed by qPCR in HAoECs at 8, 24 and 48 h after a co-culture with miRNA-709-encapsulating EVs. miR-709 expression levels were normalized by miR-186-5p (n = 3; unpaired t-test). (**B**, **C)** The expression levels of the FGF2 and the BMX mRNAs were analyzed by qPCR in HAoECs at 8 and 24 h after a co-culture with miRNA-709-encapsulating EVs. The expression levels of the FGF2 and the BMX mRNAs were normalized by ACTB (n = 3; unpaired t-test).

Finally, in order to observe whether the expression levels of the FGF2 mRNA were increased after cells were treated with miR-709-EVs, the expression levels of the FGF2 mRNA were analyzed after the addition of miR-709-EVs to HAoECs. Although the miR-709 expression level was only 1.2-fold higher in miR-709-EVs-treated HAoECs (as compared to that of scramble-EVs-treated HAoECs) at 8 h after the addition of the EVs, the miR-709 expression levels were more than 2.6-fold higher in miR-709-EVs-treated HAoECs (as compared to those of scramble-EVs-treated HAoECs) at 24 and 48 h after the addition of the EVs (Fig. 4A). Although the FGF2 and the BMX expression levels in miR-709-EVs-supplemented HAoECs were almost the same as in scramble-EVs-treated HAoEC, the mRNA expression levels were found to be 2.6- and 5.6-fold higher in miR-709-EVs-treated HAoECs (as compared to those of the scramble-EVs-treated HAoECs) at 24 h after the addition of the EVs (Fig. 4B,C).

### miR-709 bound to the 3′UTR of the human GSK3B followed by an upregulation of FGF2

**Figure 5:**
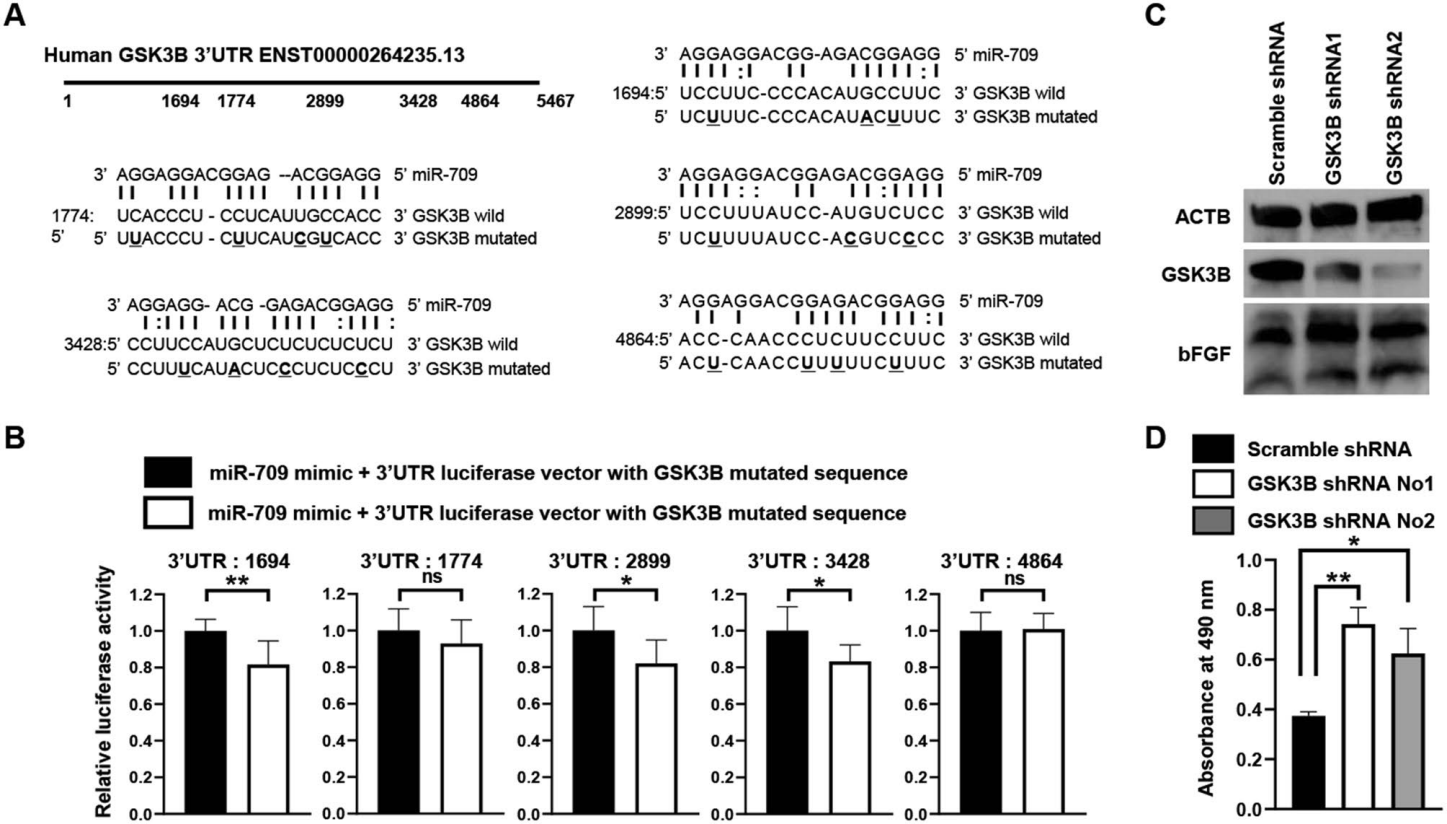
miR-709 bound to the 3′UTR of the human GSK3B followed by an upregulation of FGF2. (**A)** Five human GSK3B 3′UTR sequences (ENST00000264235.13) and the complementary miR-709-binding sequences. The miR-709-binding sites in the five sites of GSK3B 3′UTR were predicted by the RNA22 v2 miRNA target detection software. The upper sequence corresponds to miR-709, the middle one corresponds to the target wild-type of GSK3B, and the bottom corresponds to the target mutated sequence of GSK3B; underbars highlight the mutated sites. (**B)** 3′UTR luciferase assay. The 293 T cells were transfected with a miR-709 mimic and 3′UTR vectors with wild-type or mutated-type sequences. Cell lysates were used in order to measure the relative luciferase activities, 2 days after transfection. The relative luciferase activities of the miR-709 mimic and the 3′UTR vectors with the wild-type sequence were normalized by that of the miR-709 mimic and 3′UTR vectors with the mutated-type sequence (n = 7; unpaired t-test). (**C)** GSK3B and FGF2 protein levels in HAoECs expressing GSK3B shRNA. The GSK3B knockdown was carried out by using a lentivirus transduction. GSK3B and FGF2 protein levels were analyzed by Western blotting. ACTB was used as an internal control. (**D)** Cell proliferation in HAoECs expressing GSK3B shRNA. Cell proliferation was assessed by MTS reagents at 7 days after the cell seeding (n = 3; Sidak’s multiple comparisons test).

It was predicted that miR-709 could target five potential complementary binding sites of the human GSK3B 3′UTR sequence based on an algorithm (Fig. 5A). A 3′UTR luciferase reporter assay has revealed that miR-709 can actually target three sites (3′UTR: 1694, 2899 and 3428) within the human GSK3B 3′UTR sequence (Fig. 5B). The expression levels of the GSK3B protein were found to be downregulated in two GSK3B-knockdown HAoEC lines (as compared to the respective scramble-knockdown HAoECs) and the expression levels of the FGF2 protein were found to be upregulated in two GSK3B-knockdown HAoEC lines (as compared to the respective scramble-knockdown HAoECs) (Fig. 5C). Cell proliferation was found to be significantly increased in two GSK3B-knockdown HAoEC lines, as compared to their respective scramble-knockdown HAoECs (Fig. 5D). In miR-709-EVs-treated HAoECs, GSK3B protein expression was downregulated, whereas FGF2 was upregulated versus their respective scramble-EVs-treated HAoECs (Supplementary Fig. 5). EVs isolated from the medium were transfected directly with miR-709. The amount of miR-709 was approximately 46,000-fold higher in miR-709-transfected EVs compared with the scrambled-transfected EVs (Supplementary Fig. 6A). GSK3B protein expression was downregulated in miR-709-EVs-treated 293 T cells (Supplementary Fig. 6B, C).

## Discussion

In this study, we screened for angiogenic miRNAs by studying the differences in the angiogenic response between C57BL/6 and BALB/c mice undergoing hindlimb ischemia^14^. Although angiogenesis-related miRNAs have been previously selected by analyzing the miRNAs’ expression in the muscle tissues of the ischemic hindlimb^14,15,^ it is not the case for the miRNAs’ expression in miRNA-contained EVs isolated from the plasma. Moreover, this paper is the first to report that miR-709 is identified as an angiogenic miRNA after a three-stage screening (Fig. 1). The sequences of human and mouse miRNAs registered at the miRBase (https://www.mirbase.org/) are 1,917 and 1,234, respectively. Therefore, mature miRNAs often exist in both humans and mice. Although miR-709 is coded in the mouse chromosome 8q, it is not coded in the human chromosome. It is considered that miR-709 is one of the unique miRNAs. C57BL/6 mice have been used for the experimental simulation of hindlimb ischemia by many studies. Those studies also show that the blood flow in the ischemic hindlimb recovered as the time passed after the ligation of the femoral artery^16–20.^ However, the blood flow did not recover in the ischemic hindlimb of the BALB/c mice^21,22^.

In this study, we chose EVs as a delivery method of miRNAs. In order to produce miRNA–encapsulating EVs, miRNAs were transfected into 293 T cells that exhibit a high transfection efficiency and can be cultured by using a synthetic medium. EVs were isolated by using a size exclusion chromatography column from a conditioned medium in 293 T cells after the miRNA transfection. The characteristics of isolated EVs were analyzed with reference to the MISEV2014 guidelines (Fig. 2)^23^. The isolated EVs bear CD9 and CD81 (that are EV markers), but do not bear GM130 (that is a Golgi apparatus marker). The mean diameters of the isolated EVs ranged between 209 and 243 nm, and the isolated EVs were classified as lEVs with reference to the MISEV2018 statement^11^.

Animals of young age are commonly used in most animal models due to convenience. Most studies use 6–20-week-old mice for the simulation of hindlimb ischemia^16–22^. Pursuing a model that is as close to clinical practice as possible, young mice were not used in our hindlimb ischemia model (Fig. 3C). The isolated EVs contained large amounts of miRNAs by transfecting the latter into 293 T cells (Fig. 3A,B). The isolated EVs including miRNA-709 were able to upregulate the expression levels of the FGF2 mRNA in the thigh tissues of 14-month-old BALB/c mice suffering from hindlimb ischemia (Fig. 3C). In fact, 14-month-old mice correspond to the late 40 s humans based on the lifespans of the two species^24^. The FGF2 was used as an index of angiogenesis because the widths of the capillary walls were found to be larger after an FGF2 injection as compared to those identified after a VEGF injection by the mouse corneal angiogenesis assay^25^, while the injection of an FGF2-expressing vector has exhibited high limb survival in the auto-amputation model using BALB/c nu/nu mice^26^. We thought that it would be essential to activate the endothelial cells that form the arteries so as to improve the blood flow in the ischemic tissue. miR-709 was directly transfected into artery-derived endothelial cells and the expression levels of the FGF2 protein were upregulated in HAoECs (Fig. 3D). These results indicate that the blood EVs including miR-709 improved the blood flow recovery observed in the third screening (Fig. 1C). Although miR-709 is not encoded in the human chromosome, it was detected in scramble-transfected 293 T cells and scramble-encapsulating EVs (Fig. 3A), which we consider to be a non-specific detection. A previous kit (i.e., TaqMan microRNA reverse transcription kit (Thermo Fisher Scientific)) required specific RT primers for each microRNA to synthesize cDNAs from the microRNA samples. To detect microRNAs using TaqMan advanced miRNA assays (Thermo Fisher Scientific), we used the TaqMan™ advanced miRNA cDNA synthesis kit (Thermo Fisher Scientific) for miRNA-to-cDNA synthesis in this study. This cDNA synthesis kit proved to be extremely useful for analyzing multiple targets as it allowed for the simultaneous synthesis of multiple cDNAs from multiple miRNAs. In this cDNA synthesis kit, cDNA amplification occurs in four steps. Therefore, we beleive that non-specific synthesis may have been detected.

The miR-709 expression was upregulated in HAoECs at 24 h after a co-culture with EVs (Fig. 4A). The results suggested that the EVs including the miR-709 were incorporated into the HAoECs. Although the expression levels of the FGF2 mRNA were the same in the HAoECs at 8 h after a co-culture with scramble- or miR-709-encapsulating EVs, the expression levels of the FGF2 mRNA were found to be upregulated in HAoECs at 24 h after a co-culture with EVs including the miR-709, as compared with those of HAoECs co-cultured with scramble-encapsulating EVs (Fig. 4B). The data were consistent with the FGF2 expression levels found in vivo 2 days after an injection of miR-709-containing EVs (Fig. 3C). These data suggest that the FGF2 expression was induced by the miR-709 that was transferred from the EVs into the cells. It has been reported that the non-receptor tyrosine kinase BMX is one of the markers of arterial endothelial cells^27^, and that the expression levels of the BMX mRNA are much higher in endothelial stem cells than in non-endothelial stem cells.^28^ The expression levels of the BMX mRNA were found to be upregulated at 24 h after a co-culture with EVs containing miR-709 (Fig. 4C); a finding suggesting that miR-709 may have an ability to activate the arterial endothelial cells.

In an attempt to elucidate the link between miR-709 and FGF2, we explored the miR-709 target genes that are related to FGF2. It has been reported that the Wnt pathway activates the FGF2 in fibroblasts^29^, and that miR-709 can target in one place the 3′UTR of GSK3B, followed by an activation of the Wnt signaling pathway (Supplementary Fig. 4)^30^. The study reported that miR-709 had one binding site for the 3′UTR of the mouse and the human GSK3B, as predicted by TargetScan (https://www.targetscan.org/vert_80/). In order to predict the 3′UTR of the GSK3B targeted by miR-709, we used the RNA22 v2 miRNA target detection software instead of TargetScan. A target scan algorithm (RNA22 v2 miRNA target detection) predicted that miR-709 might have five binding sites for the 3′UTR of the human GSK3B (Fig. 5A) and six binding sites for the 3′UTR of the mouse GSK3B (Supplementary Fig. 3). Our study showed that miR-709 actually had three binding sites for the 3′UTR of the human GSK3B (Fig. 5B). The knockdown of the GSK3B and EVs containing miR-709 increased the cell growth and the FGF2 expression in HAoECs (Fig. 5C,D, Supplementary Fig. 5). Our data suggested that the miR-709-encapsulating EVs were incorporated into the endothelial cell, and that miR-709 downregulated the GSK3B protein, thereby allowing for the proliferation of the endothelial cells. EVs that were directly transfected with exhibited downregulated GSK3B protein in 293 T cells (Supplementary Fig. 6B,C). This experiment was conducted in 293 T cells because cell death was observed three days after addition of miRNA mimic in transfected EV-treated HAoEC cells. Although it has been reported that miR-709 does not inhibit GSK3B in mouse primary hepatocytes^31^, it does inhibit GSK3B in mouse 3T3-L1 cells^30^. It is also known that miR-709 silences EGR2 by binding to the 3′UTR and the promoter region of EGR2^32^. Meanwhile, EGR2 is expressed in venular endothelial cells, but not in the endothelial cells of arterioles and post-arterial capillaries, based on an analysis of single-cell transcriptomic profiling for human dermal blood endothelial cells^33^. These suggest that miR-709 is expressed in arterial, but not in venous, endothelial cells. It has also been reported that miR-709 expression levels increase over time from postnatal day 0–21 during mouse retinal development^34^. It is also predicted that miR-709 targets SEMA3F, an angiogenic inhibitor^35^. These findings suggest that miR-709 may inhibit the functions of angiogenesis inhibitory genes.

In this study, we demonstrated that miR-709 is an angiogenic miRNA. miR-709-encapsulating EVs recovered the blood flow in hindlimb ischemic model BALB/c mice and increased the expression levels of FGF2 mRNA in their thigh tissues. Co-culturing with miR-709-encapsulating EVs also increased the expression levels of FGF2 and BMX mRNAs in HAoECs. The binding of miR-709 to the 3′UTR of GSK3B caused an upregulation of the FGF2 expression and an increase of the proliferation of endothelial cells. Therefore, our results suggested that miR-709 might be a promising angiogenic agent for the treatment of CLI.

## Methods

### Animals

All animal procedures were approved by the Institutional Animal Care and Use Committee of Yamaguchi University (No. 31-101). The study was conducted in accordance with the relevant guidelines and carried out in compliance with the ARRIVE guidelines. The methods were carried out in accordance with the approved guidelines and the ARRIVE guidelines. Male C57BL/6 and male BALB/c mice were purchased from Japan SLC, Inc. (Shizuoka, Japan). Five mice were housed in the same cage (mouse polycarbonate cage, #TM-PC-5-I(1), Tokiwa, Tokyo, Japan) Fir wood shavings were used as beddings. Mice were bred in a temperature-, humidity-, and light-controlled sub-SPF room (22 ± 2 °C, 70 ± 20%, and 12 h light/dark cycles, respectively) at Institute of Life Science and Medicine Yamaguchi University. Food and water were provided ad libitum. Mice were randomly divided into each group. Anesthesia was maintained with 1.5–2% isoflurane (MSD Animal Health, Tokyo, Japan).

### miRNA extract and array analysis

Mice were anesthetized through the inhalation of 1.5% isoflurane during the surgical procedure. For the first screening, the left femoral artery and vein in 8-week-old C57BL/6 and BALB/c mice was ligated at two points, and was severed with an electric scalpel between the ligated points. In order to collect plasma at 24 h after the ligation of the left femoral artery, blood was drawn from the abdominal vena cava by using a 26-G syringe including 0.1 mL of 1% ethylenediaminetetraacetic acid (EDTA). The collected blood was transferred to a 1.5-mL tube, and was centrifuged at 1,200 g for 20 min, at 4 °C. The supernatant was placed in a new 1.5-mL tube, and was stored at −80 °C. EVs were isolated from the obtained plasma by using the total exosome isolation kit (#4484450; Thermo Fisher Scientific, Waltham, Massachusetts, USA). miRNAs in the EVs were extracted with the use of a total exosome RNA and protein isolation kit (#4478545; Thermo Fisher Scientific). The miRNA expression levels were analyzed in each of the five-sample pools (n = 5 in C57BL/6, and n = 5 in BALB/c) by using 3D-Gene® miRNA oligo chips (Toray Industries, Inc., Kamakura, Kanagawa, Japan) (GEO accession: GSE217938 and Supplementary Data).

### Plasmids expressing miRNAs

In order to construct the plasmids expressing the 43 miRNAs shown Supplementary Table 1, the C57BL/6 mouse genome was amplified with Ex Taq (TaKaRa Bio Inc., Kusatsu, Shiga, Japan) and primers (Supplementary Table 1), and the amplified DNA was ligated to a T-vector pMD20 (#3270; TaKaRa Bio Inc). Primers were designed with reference to mice chromosome region (Supplementary Table 1). DNA fragments were subcloned from the T-vector pMD20 into the restriction enzymes’ (Supplementary Table 1) site of the pmR-mCherry vector (#Z2542N; TaKaRa Bio Inc).

### Preparation of miRNA-containing EVs for the aortic ring assay

293 T cells (#RCB2202) were provided by the RIKEN BRC through the National Bio-Resource Project of the MEXT/AMED, Japan. The cells were cultured in Dulbecco’s modified Eagle medium (DMEM; #11995-065; Thermo Fisher Scientific) supplemented with 10% exosome-depleted fetal bovine serum (FBS; #A2720801; Thermo Fisher Scientific), and 0.5 mL of 293 T cells (2 × 10^5^ cells/mL) were seeded in 24-well plates. Subsequently, the cells were transfected with 1 μg of the plasmid by using 1 μL of the X-tremeGENE HP DNA transfection reagent (Roche), and were incubated overnight. After removing the culture medium from the wells, 1.2 mL/well of fresh DMEM supplemented with 10% exosome-depleted FBS was applied to the same wells. The culture medium was collected after 3 days. The EVs included in the culture medium were isolated by using the total exosome isolation reagent (from cell culture media; #4478359; Thermo Fisher Scientific). Finally, the pellets were dissolved with 420 μL of Opti-MEM™ I reduced serum medium (Thermo Fisher Scientific).

### Aortic ring assay

An aortic ring assay was carried out based on previous reports^36,37.^ Femoral arteries were removed from both thighs of BALB/c mice, and were immersed in phosphate-buffered saline (PBS) on ice. At first, 50 μL of Matrigel (Cornig) were applied on 44 wells of a 96-well plate that was plated on ice. An aortic ring was placed on the Matrigel of each well. Subsequently, 50 μL of Matrigel were added in each well, and the 96-well plate was incubated at 37 °C, in 5% CO2, overnight. The next day, 50 μL of the EVs’ solution were applied on each well, and the 96-well plate was incubated for 5–7 days at 37 °C, in 5% CO2. Three independent experiments were performed. If neovascularization was observed under the microscope even at a rate of one out of three, the miRNA was considered to have an angiogenic potential.

### Preparation of miRNA-containing EVs for screening by using the hindlimb ischemia model

293 T cells were seeded in 6-well plates, and were cultured in DMEM supplemented with 5% exosome-depleted FBS. Cells were transfected with 2 μg of plasmid by using 2 μL of X-tremeGENE HP DNA transfection reagent (Roche), and were incubated overnight. After removing the culture medium from the wells, 2 mL/well of fresh DMEM supplemented with 5% exosome-depleted FBS were applied to the wells. The culture medium was collected after 5 days. The EVs included in the culture medium were isolated by using the total exosome isolation reagent. The pellets were then dissolved with 400 μL of PBS.

### Screening of angiogenic miRNAs by using a lower ischemia model

The left femoral artery was ligated in one place in 7-week-old BALB/c mice. The next day, a total of 100 μL of EVs were injected in two places in the femoral muscle. Blood flow was analyzed by using a laser speckle perfusion imaging system (OMEGA ZONE, Omega Wave). Three mice per one group in one experiment were assessed. Two independent experiments were performed. Blood flow was analyzed in six mice per group.

### Preparation of miRNA-encapsulating EVs

293 T cells were cultured in HE100 medium (Gmep Incorporated, Kurume, Fukuoka, Japan) with L-glutamine. Scramble, miR-709, and miR-1247-3p mimics were synthesized by Ajinomoto Bio-Pharma Services, GeneDesign Inc. (Osaka, Japan). 293 T cells were seeded in a 10-cm dish (2 × 10^6^ cells/dish) by using 10 mL of HE100 medium supplemented with L-glutamine. The next day, the 293 T cells were transfected with 600 pmol of miRNA mimics by using 25 μL of the RNAiMAX transfection reagent (#13778075; Thermo Fisher Scientific), and were incubated for 24 h after transfection. After removing the culture medium from the dishes, 10 mL/dish of fresh HE100 medium supplemented with L-glutamine was applied to the dishes. The culture medium was collected after 3 days. The culture medium was concentrated with Amicon Ultra-15 (#UFC901024, Merck Millipore Ltd.), and the concentrated solution was applied to a qEV2 70 column (Meiwafosis Co., Ltd., Tokyo Japan) in order to isolate the EVs. The EV concentration was measured by using a CD9/CD63 exosome ELISA kit (#EXH0102EL; Cosmo Bio Co., Ltd., Tokyo, Japan).

### Introducing miRNAs directly into EVs

293 T cells were cultured in HE100 medium with L-glutamine. The culture medium was concentrated using an Amicon Ultra-15 and the concentrated solution was applied to a qEV2 70 column to isolate the EVs. The EV concentration was measured using a CD9/CD63 exosome ELISA kit. Scrambled or miR-709 mimics were directly transfceted into EVs using the Exo-Fect siRNA/miRNA Transfection Kit (#EXFT200A-1, SBI). 293 T cells (5 × 10^4^ cells/2 mL/well) were seeded into 6-well plates in DMEM with 5% FBS and cultured overnight. 293 T cells were incubated with scrambled or miR-709-EVs overnight and cultured for 2 days after a medium exchange. Proteins were extracted three days after culture with the addition of scrambled or miR-709-EVs.

### Western blotting for EVs

EVs (1,000 pg/ 300 μL) were pelletized by using the total exosome isolation reagent. The pellets were dissolved with 50 μL of 4 × Laemmli sample buffer (#1610747, Bio-Rad). Subsequently, 10 μL of each sample (200 pg EVs) were applied to each well and were subjected to Western blotting by using a CD9 monoclonal antibody (Ts9; 1:500; #10626D; Thermo Fisher Scientific), a CD81 monoclonal antibody (M38; 1:500; #10630D; Thermo Fisher Scientific), a GM130 (D6B1) XP® rabbit monoclonal antibody (1:500; #12480; Cell Signaling Technology), a goat anti-mouse Ig/HRP (affinity isolated) antibody #P0447; Dako, Glostrup, Denmark), and a goat anti-rabbit Ig/HRP (affinity isolated) antibody (1:2,000; #P0448; Dako). Extracted proteins were visualized by using the ECL™ prime Western blotting detection reagent (#RPN2232; Cytiva, Marlborough, MA, USA). The images were detected by using Amersham™ Imager 600 (GE Healthcare). Precision Plus Protein™ Dual Color Standards was used as a marker (#1610374, Bio-Rad).

### EV size distribution and morphology

The EV size distribution was analyzed by using LM10 NanoSight (Malvern Instruments, UK). The relevant results are shown by standard error (n = 5). The morphology of the EVs was observed with a transmission electron micrograph.

### Analysis of miRNA expression levels in miRNA-encapsulating EVs and miRNA directly -transfected EVs

miRNAs were extracted from cells and EVs with the use of miRNeasy mini kits (#217004; Qiagen) and the total exosome isolation reagent. cDNAs were synthesized from miRNAs with the use of the TaqMan™ advanced miRNA cDNA synthesis kit (#A28007; Thermo Fisher Scientific), and qPCR was performed by using the cDNAs with a TaqMan™ fast advanced master mix (Thermo Fisher Scientific) and the following TaqMan advanced miRNA assays (Thermo Fisher Scientific): hsa-miR-186-5p (477940_mir), mmu-miR-709 (mmu482967_mir), and mmu-miR-1247-3p (mmu480903_mir). Quantitative PCR was performed on a StepOnePlus instrument (Thermo Fisher Scientific), and the employed quantitative PCR parameters for cycling were as follows: 95 °C for 20 s, 40 cycles of PCR at 95 °C for 1 s, and 60 °C for 20 s. All reactions were undertaken in 20-μL reaction volumes, in triplicate. The miRNA expression levels were determined by using the 2^–ΔCT^ method.

### Analysis of the levels of the FGF2 mRNA after injecting miR-709-containing EVs in hindlimb ischemia model mice

The left femoral artery in 14-month-old BALB/c mice was ligated in one place. At 4 days after the ligation of the left femoral artery, a total of 100 μL of EVs (100 pg) were injected in two places in the femoral muscle. Two days after injecting the EVs, the femoral muscles were removed and were stored at 4 °C, overnight, after being immersed in the RNAlater™ stabilization solution (#AM7020; Thermo Fisher Scientific). Total RNAs were extracted from the femoral muscles with the use of the RNeasy mini kit (#74104; Qiagen). The extracted total RNA was then reverse-transcribed into single-stranded cDNA by using the PrimeScript™ RT reagent kits (Perfect Real Time; TaKaRa Bio Inc.), and qPCR was performed by using cDNAs with the SYBR™ select master mix (#4472918; Thermo Fisher Scientific). The primer sequences used are summarized in Supplementary Table 2. Quantitative PCR was performed on a StepOnePlus instrument (Thermo Fisher Scientific). Quantitative PCR parameters for cycling were as follows: 50 °C for 2 min followed by 95 °C for 2 min, 40 cycles of PCR at 95 °C for 3 s, and 60 °C for 30 s. All reactions were performed in 10-μL reaction volumes, in triplicate. The mRNA expression levels were determined by using the 2^–ΔCT^ method.

### FGF2 measurement using ELISA in miRNA-transfected Human aortic endothelial cells (HAoEC)

Human aortic endothelial cells (HAoECs; #C-12271; TaKaRa Bio Inc.) were seeded to 6-well plates (1 × 10^5^ cells in 2 mL/well), and were cultured with (ready-to-use) endothelial cell growth medium MV 2 (#C-22022; TaKaRa Bio Inc) overnight. Subsequently, 100 pmol of miRNA mimics were transfected to the HAoECs, and were left to incubate for 3 days. A total of 300 μL of cold buffer containing 10 mM of Tris–HCl (pH 7.4), 0.5 mM of egtazic acid, 0.5 mM of EDTA, 1% Triton X-100, and cOmplete™ mini protease inhibitor cocktail (Sigma, St Louis, MO, USA) were applied to each well, and the cells were incubated for 30 min on ice and then centrifuged at 13,000 rpm for 20 min, at 4 °C. The supernatants were collected, and their protein concentration was measured by using a Pierce™ BCA protein assay kit (#23227; Thermo Fisher Scientific). After that, 1 μg of protein was applied to each well and the FGF2 concentration was measured by using a human FGF basic/FGF2/bFGF Quantikine HS ELISA kit (#HSFB00D; R&D Systems, Inc., Minneapolis, Minnesota, USA).

### FGF2 mRNA expression after the addition of miR-709-EVs in HAoECs

HAoECs were seeded in 6-well plates (6 × 10^4^ cells in 2 mL/well) and were incubated overnight. Subsequently, 1,000 pg of scramble- or miR-709-EVs were added in each well, and were incubated for 8, 24, and 48 h. The expression levels of miR-709, FGF2, and BMX were analyzed by the method described above.

### 3′UTR luciferase reporter assay

A pmirGLO dual-luciferase miRNA target expression vector (#E1330; Promega) was used for the 3′UTR luciferase assays. The 3′UTR of the human and the mouse GSK3B sequences targeted by miR-709 were predicted based on the RNA22 v2 miRNA target detection software (https://cm.jefferson.edu/rna22/Interactive/). Supplementary Table 3 summarizes the oligo sequences used for constructing the 3′UTR plasmids for the human GSK3B. The plasmids for the 3′UTR luciferase assays were constructed as described previously^38^. For the 3′UTR luciferase assay, 293 T cells were seeded to 48-well plates (2 × 10^4^ cells in 0.2 mL/well) and were cultured at 37 °C, in 5% CO2, overnight. 293 T cells were then transfected with 40 pmol of the miR-709 mimic and 200 ng of pmirGLO dual-luciferase miRNA target expression vectors with wild-type or mutated target sequences by using 0.67 μL of lipofectamine 2000 (Thermo Fisher Scientific) for each well of a 48-well plate. The luciferase assay was performed at 2 days after transfection by using the dual-luciferase® reporter assay system (#E1910; Promega). The recombinant DNA research was approved by the Genetic Modification Safety Committee of Yamaguchi University (#J20003).

### FGF2 and GSK3B protein expression and cell proliferation assay in GSK3B-knockdown HAoECs and miR-709-EVs-treated HAoECs

For GSK3B-knockdown HAoECs, a pSIH1-H1-Puro shRNA cloning and expression vector (#SI500A-1; SBI) was used for the target gene knockdown. Target sequences for knockdown were predicted based on siDirect version 2.0 (http://sidirect2.rnai.jp/) data. Supplementary Table 4 presents the target sequences and the oligo sequences used for the construction of the plasmids. 293 T cells were seeded in 10-cm dishes and were transfected with 6 μg of the pSIH1-H1-Puro shRNA cloning and expression vector and with 4 μg of the pPACKH1 lentivector packaging kit (#LV500A-1; SBI) by using 10 μL of the X-tremeGENE HP DNA transfection reagent (Roche) for constructing pseudoviral particles. HAoECs were incubated with the pseudoviral particles and the TransDux MAX lentivirus transduction enhancer (#LV860A-1; SBI) for 3 days, and were cultured under puromycin dihydrochloride (#A1113803; Thermo Fisher Scientific) for 2 days followed by a further culturing of the cells for the undertaking of the proliferation assay. For miR-709-EVs-treated HAoECs, cells (1 × 10^4^ cells in 2 mL/well) were seeded in a 6-well plate and incubated overnight. Subsequently, 500 pg of scramble- or miR-709-EVs were added in each well and incubated for 3 days. For the determination of the FGF2 and GSK3B protein expression, the protein was extracted by the method described above. Subsequently, 30 μg of the sample were applied to each well and were subjected to Western blotting by using a β-actin (13E5) rabbit monoclonal antibody (1:1,000; #4970; Cell Signaling Technology, Inc.), a GSK-3β (27C10) rabbit monoclonal antibody (1:1,000; #9315; Cell Signaling Technology, Inc.), an anti-FGF2 (EPR20145-219) antibody (1:1,000; #ab208687; Abcam), and a goat anti-rabbit Ig/HRP (affinity isolated) antibody (1:2,000; Dako). For the undertaking of the cell proliferation assay, the cells (5 × 10^3^ cells in 0.2 mL/well) were seeded into a 96-well plate and were incubated for 7 days. Cell proliferation was evaluated by absorbance measurements at 490 nm, through the use of a 2030 ARVO X4 (PerkinElmer, Boston, MA, USA) after using the CellTiter 96® AQueous One Solution Cell Proliferation Assay kit (#G3580, Promega) according to the manufacturer’s instructions.

### Statistical analysis

All statistical analyses were performed by using the GraphPad Prism 8 software (GraphPad Software, USA). A p-value of <0.05 was considered as indicative of statistically significant differences.

### Ethical approval and consent to participate

All animal procedures were approved by the Institutional Animal Care and Use Committee of Yamaguchi University (#31-101). The methods were carried out in accordance with the approved guidelines.

### Supplementary Information

Supplementary Information.

## Data Availability

The datasets generated and/or analysed during the current study are available in the GEO repository (GEO accession: GSE217938).
