# Supplementary material for: miR-709 exerts an angiogenic effect through a FGF2 upregulation induced by a GSK3B downregulation
Source: Sci Rep. 2024 May 18;14:11372. doi: 10.1038/s41598-024-62340-4 (PMC11102560; doi:10.1038/s41598-024-62340-4)
Supplement: Supplementary file 1 — Supplementary Information. [file 41598_2024_62340_MOESM1_ESM.pdf]

Supplementary Figure 1

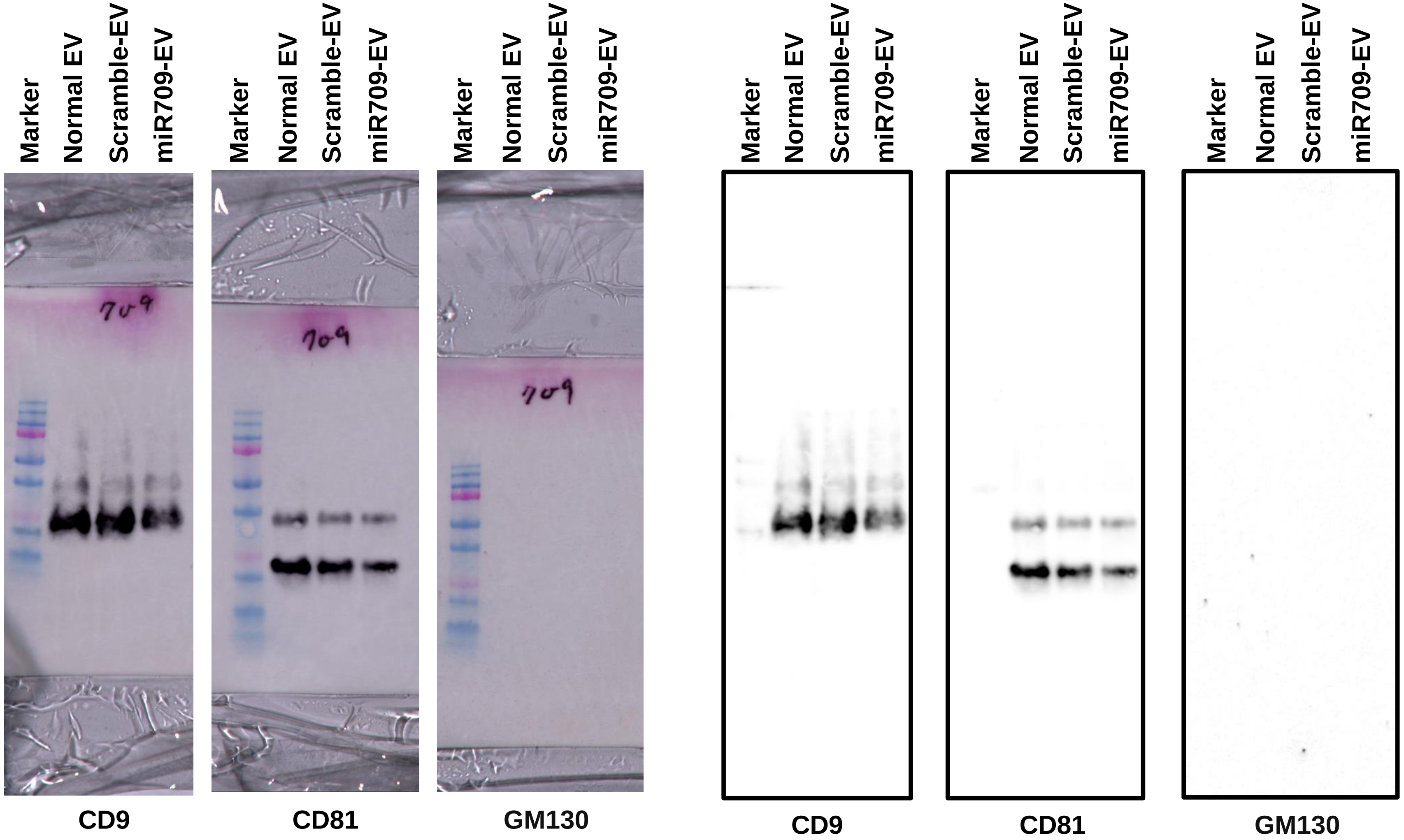

Supplementary Figure 2

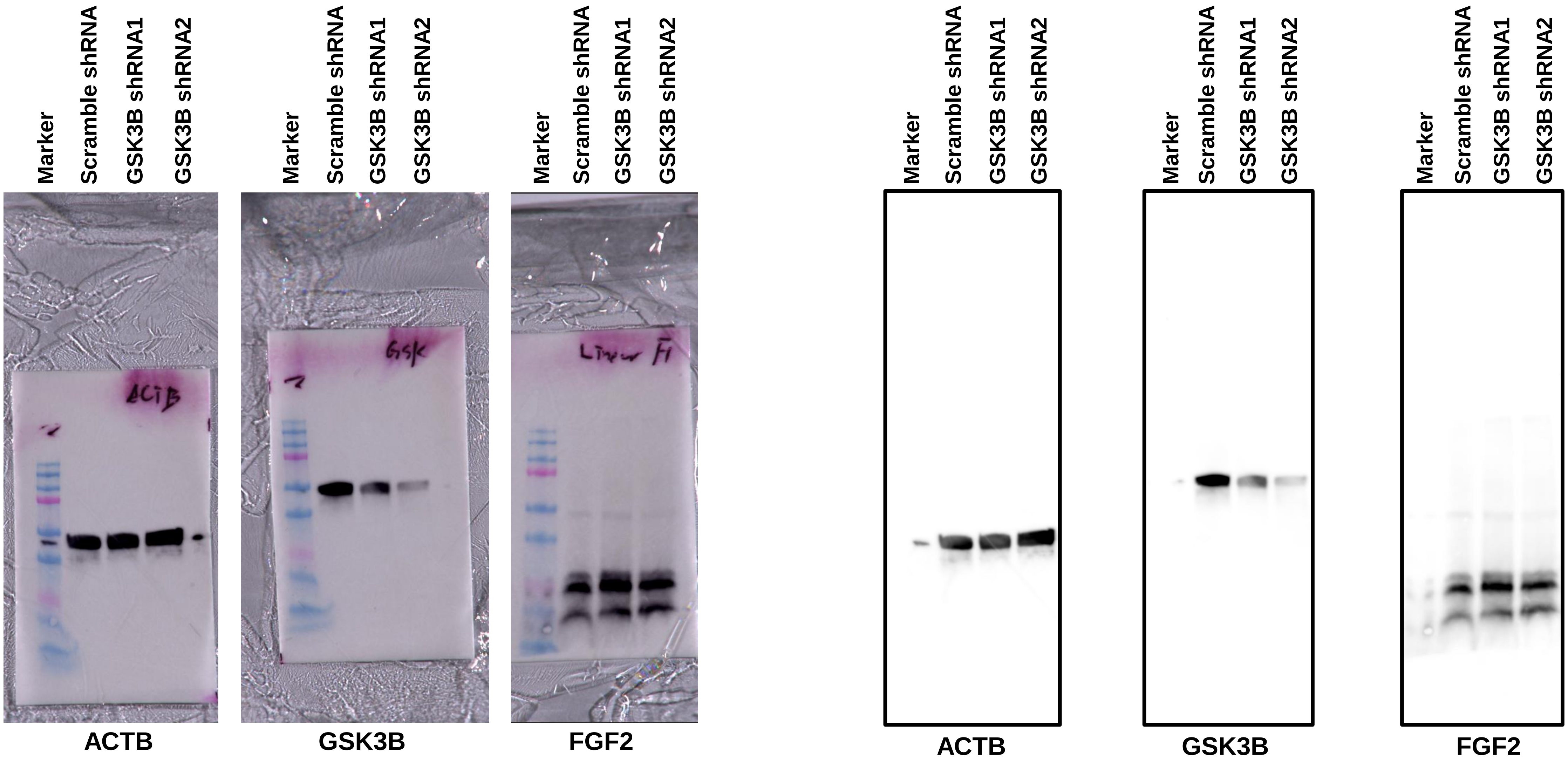

Supplementary Figure 3

Mouse GSK3B 3'UTR ENSMUST00000023507.13

1 1333 1714 1894 1900 3522 4232 5515

```
        3'  AG  -GAGGACGGAGACGGAGG   5' miR-709
            ||      ||||||:|  |:| ::
1333:       UCAAUACUGCCUUUUCUUUU   3' GSK3B wild
5'

          3' AGGA-GGA-CGGAGACGGAGG   5' miR-709
             |||| |||  ||  ||  |||||
   1714:5'   UCCUUCCUUCCCACUCCCUCC   3' GSK3B wild

         3'   AGGAGGACG-GAGACGGAGG   5' miR-709
                || |: || |  ||||||:
   1894:5'    GCC-CUAGCACCCUGCCUUG   3' GSK3B wild

          3'  AGGA  GGACGGAGACGGAGG   5' miR-709
                |    |||||||  |||:||:
   1900:5'    GCACCCUGCCU-UGCUUCU   3' GSK3B wild

          3' AGG-  -AGGACGGAGACGGAGG   5' miR-709
             ||:     || |:   ||  |||||
   3522:5'   UCUGAGCCCGUUCCUCCCUCC   3' GSK3B wild

          3' AGGAGG-  ACGGAGACGGAGG   5' miR-709
                || ||   ||       |:||||
   4232:5'   GCC-  CCAGGCAGAGGUCUCC   3' GSK3B wild
```

Supplementary Figure 4

```
    3' AGGAGGACGGAGACGGAGG   5' miR-709
                  |||||||
342:5' AAACCAGACUCCUGCCUCA   3' GSK3B mouse
324:5' AAACAGGACUCCUGCCUCA   3' GSK3B human
```

Supplementary Figure 5

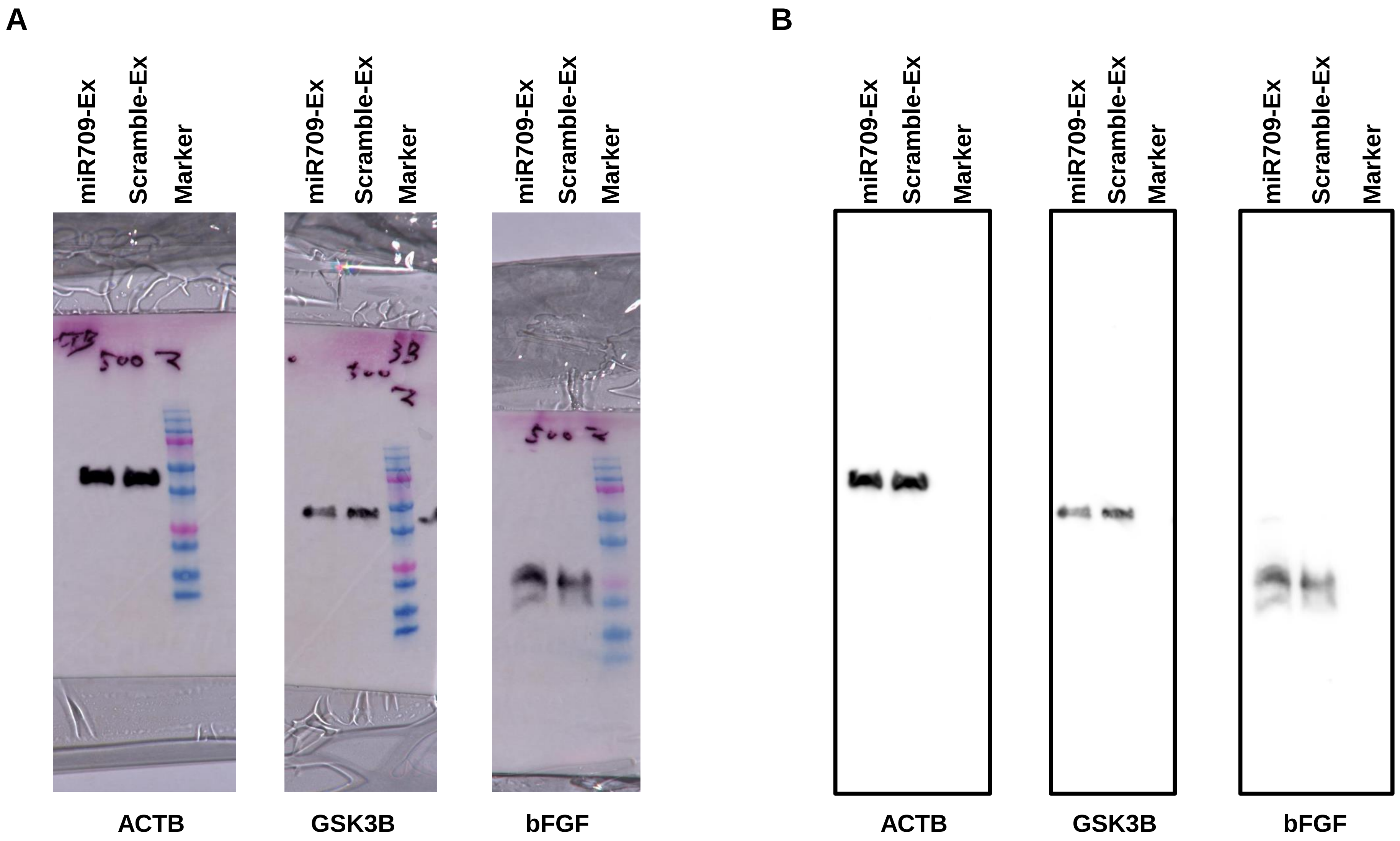

Supplementary Figure 6

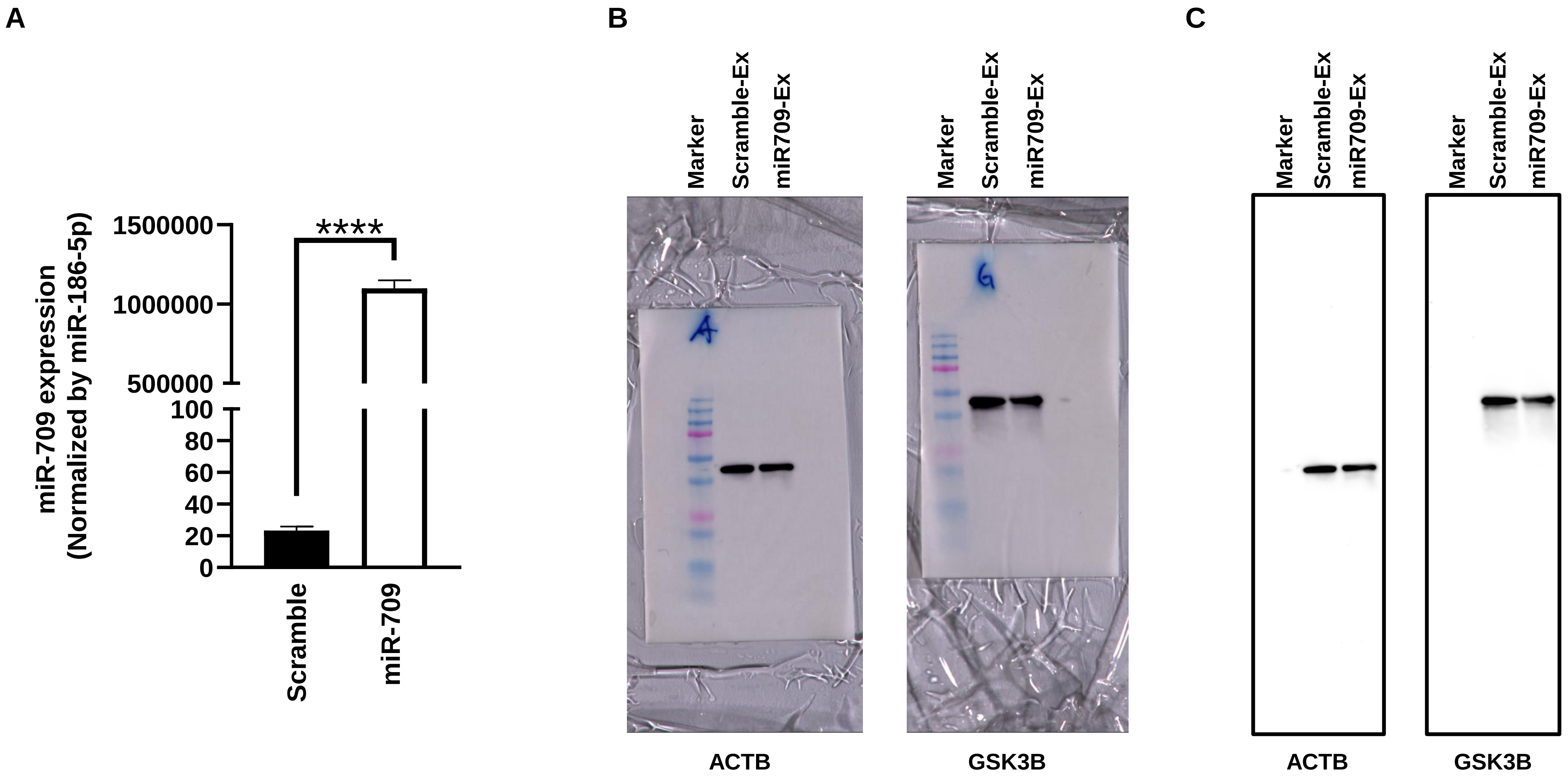

Supplementary Figure legends

Supplementary Figure 1

Raw data for Figure 2A. These figures represent full-length blots with and without colorimetric markers (left and right sides). The exposure was captured in the automatic mode.

Supplementary Figure 2

Raw data for Figure 5C. These figures represent full-length blots with and without colorimetric markers (left and right sides). Figure 5C was excised from the western blot data with the colorimetric markers presented in this figure (Supplementary Figure 2); however, Figure 5C did not undergo any image processing. The exposure in the figures on the right was captured in automatic mode without colorimetric markers.

Supplementary Figure 3

Mouse GSK3B 3′-UTR sequences (ENSMUST00000023507.13) and complementary miR-709-binding sequences. The miR-709-binding sites at six sites in the mouse GSK3B 3′-UTR were predicted using RNA22 v2 miRNA target detection. The upper sequence corresponds to that of miR-709, whereas the lower sequence corresponds to the target sequence of mouse GSK3B.

Supplementary Figure 4

miR-709-binding sites in the mouse and human GSK3B 3′-UTR, as predicted by TargetScan. The upper sequence corresponds to miR-709, the middle sequence is the mouse 3′-UTR sequence of GSK3B, and the bottom sequence is the human 3′-UTR sequence of GSK3B.

Supplementary Figure 5

miRNA-709-encapsulating EVs upregulate GSK3B and FGF2 expression in HAoECs. GSK3B and FGF2 protein levels were measured by western blot analysis in HAoECs three days after co-culture with miRNA-709-encapsulating EVs. ACTB was used as an internal control. A and B: GSK3B and FGF2 protein levels in HAoECs three days after co-culture with miRNA-709-encapsulating EVs. A: Full-length blots with colorimetric markers are shown. B: Full-length blots without colorimetric markers.

Supplementary Figure 6

EVs directly transfected with miR-709 downregulated GSK3B protein in 293T cells. A: miRNA mimics were directly transfected into EVs isolated from the supernatant of 293T cells, and the expression of miRNA mimics was analyzed by qPCR. The miR-709 expression levels were

normalized to those of miR-186-5p. B: Full-length blots with colorimetric markers. C: Full-length blots without colorimetric markers. GSK3B protein levels were measured using western blot analysis in 293T cells. ACTB was used as an internal control.

Supplementary Table 1. Sequences of the miRNAs identified by the first screening.

Number	miRNA<br>accession number<br>chromosome range	Primer sequence 5’ → 3’	Enzyme	PCR product	2nd screening
No1	mmu-miR-135a-1-3p	GAATTCtcagtcttaggaacacacacaggag	EcoRI	294	0
	MIMAT0004531	GGATCCagccctcttgttccacatcagag	BamHI		
	chr9:106153824-106154513				
No2	mmu-miR-365-1-5p	GAATTCtcctttttagttttggctctctgtg	EcoRI	286	2
	MIMAT0017077	GGATCCatgtgccatctttttattcaccact	BamHI		
	chr16:13453540-13454226				
No3	mmu-miR-665-3p	CTCGAGgatggggtctttgttactagagcag	XhoI	294	0
	MIMAT0003733	GAATTCtcattgtgctactgaagtgtctgtg	EcoRI		
	chr12:109586014-109586707				
No4	mmu-miR-677-3p	GAATTCagagatgagtgttgagcaggagatt	EcoRI	279	0
	MIMAT0017246	GGATCCccaaattaaacagccttcaaaaatg	BamHI		
	chr10:128084986-128085663				
No5	mmu-miR-744-5p	GAATTCatgtgtgctcattacatttttgctg	EcoRI	295	0
	MIMAT0004187	GGATCCagagatggaataaaccaacagaagc	BamHI		
	chr11:65734433-65735132				
No6	mmu-miR-1893	GAATTCaactccggttctccatgatcc	EcoRI	238	0
	MIMAT0007879	GGATCCagttaacacgtaccgactcctcttc	BamHI		
	chr18:6490264-6490946				
No7	mmu-miR-2136	GAATTCgggaattttgtggtttagattttcc	EcoRI	285	0
	MIMAT0011212	GGATCCccagacctctatcaattgactgctt	BamHI		
	chr9:104425813-104426487				
No8	mmu-miR-3968	CTCGAGctatgcctcaggatactgcttgagt	XhoI	293	0
	MIMAT0019352	GAATTCgtcgcaacatcgtctacctgct	EcoRI		
	chr11:115447661-115448360				
No9	mmu-miR-5099	CTCGAGtttccacatctctcttccacca	XhoI	392	1
	MIMAT0020606	GGATCCccctgccttccatacacca	BamHI		
	chr12:36815905-36816578				
No10	mmu-miR-5122	GAATTCgcttcctcttcttacgtgacacttc	EcoRI	269	0
	MIMAT0020630	GGATCCagcaacacacccctccttaagac	BamHI		
	chr4:133369476-133370164				
No11	mmu-miR-5131	GAATTCattataaagtccgggccgagag	EcoRI	274	1
	MIMAT0020642	GGATCCagccaggtggctcaggtc	BamHI		

	chr14:45657661-45658353				
No12	mmu-miR-5620-5p<br>MIMAT0022367<br>chr7:7298591-7299246	GAATTCgcaagtgtctgaagaagagaacagg<br>GGATCCaagtaggcgggcagtgattttct	EcoRI<br>BamHI	263	0
No13	mmu-miR-6239<br>MIMAT0024860<br>chr14:117953443-117954147	GAATTCgagcagattggtgtaaagtcaaaca<br>GGATCCtgtcttgtgtgcagtaaaaacactg	EcoRI<br>BamHI	300	0
No14	mmu-miR-6378<br>MIMAT0025124<br>chr3:34922245-34922951	GAATTCgaggctgagttaggaactgaaaaca<br>GGATCCcgagaatgaaggcagttgaatagtt	EcoRI<br>BamHI	266	0
No15	mmu-miR-6914-5p<br>MIMAT0027728<br>chr10:128382538-128383206	CTCGAGacatcaaacacagaggtccagagat<br>GAATTCatcagcaagtacctgaggagagaag	XhoI<br>EcoRI	218	0
No16	mmu-miR-6937-5p<br>MIMAT0027774<br>chr12:28679025-28679689	CTCGAGggcttggctcttgacacttttaat<br>GAATTCgaagagtaatcatgtcccccttctc	XhoI<br>EcoRI	254	0
No17	mmu-miR-6980-5p<br>MIMAT0027862<br>chr18:37990587-37991245	GAATTCagtatttgtgttccgcacagagagt<br>GGATCCctcactcttgtacagtgccagctc	EcoRI<br>BamHI	292	0
No18	mmu-miR-6987-5p<br>MIMAT0027876<br>chr19:5678704-5679376	GAATTCtctcgttttcttcctctctcttcc<br>GGATCCgctcctgtagactacccataccttg	EcoRI<br>BamHI	299	0
No19	mmu-miR-6989-5p<br>MIMAT0027880<br>chr19:6346180-6346844	GAATTCagcagaagcaataacttaggacacc<br>GGATCCcagcaacacattgttcacacagtag	EcoRI<br>BamHI	288	0
No20	mmu-miR-6991-5p<br>MIMAT0027884<br>chr19:7422273-7422942	GAATTCgaggatggaaggctgtagagagac<br>GGATCCgccgtcgtagtccatcaagtattc	EcoRI<br>BamHI	325	0
No21	mmu-miR-6999-5p<br>MIMAT0027900<br>chr2:91944570-91945230	GAATTCggcctaagaggagctacatttcatt<br>GGATCCcacatatactcacactccagtccaca	EcoRI<br>BamHI	296	0
No22	mmu-miR-7020-5p<br>MIMAT0027944<br>chr4:139643742-139644411	GAATTCggctgctttacctgaggatatgac<br>GGATCCgctgcagcgtctctctgtatttatc	EcoRI<br>BamHI	283	1
No23	mmu-miR-7042-5p<br>MIMAT0027988	GAATTCtgaggaatcggcaatgtagaaag<br>GGATCCccaacaaatcttcatcagcttcac	EcoRI<br>BamHI	218	0

	chr6:113706908-113707565				
No24	mmu-miR-7044-5p<br>MIMAT0027992<br>chr6:118084892-118085563	GAATTCatagcccactatacccatccttttc<br>GGATCCgacagcagcaagctttgtatcattt	EcoRI<br>BamHI	253	0
No25	mmu-miR-7047-5p<br>MIMAT0027998<br>chr7:24987304-24987967	CTCGAGgcaggaagcttaggcctgga<br>GAATTCagttgtcatccagcagaatcatgt	XhoI<br>EcoRI	227	0
No26	mmu-miR-7036b-5p<br>MIMAT0029808<br>chr5:34573851-34574513	GAATTCgtctaggtgcttgcctccgagt<br>GGATCCgtcagtttttaaaagagccgacaag	EcoRI<br>BamHI	237	1
No27	mmu-miR-7658-5p<br>MIMAT0029822<br>chr4:156229759-156230415	GAATTCccagcctcaactccatagagaagta<br>GGATCCgccctaacacttggtagagaagaca	EcoRI<br>BamHI	262	2
No28	mmu-miR-7662-3p<br>MIMAT0029831<br>chr10:62193867-62194527	CTCGAGgctaggcaagggagagaaaatg<br>GAATTCagatcaacagttcccacctacctg	XhoI<br>EcoRI	210	0
No29	mmu-miR-7672-5p<br>MIMAT0029850<br>chr14:26669247-26669908	GAATTCcacggtctgaatgggtcttgta<br>GGATCCctctggtccctctgcatgtttt	EcoRI<br>BamHI	299	0
No30	mmu-miR-8110<br>MIMAT0031416<br>chr8:89024435-89025131	GAATTCaatcacttcggactttctgtttgac<br>GGATCCgacctggataggagaagaagctacc	EcoRI<br>BamHI	287	1
No31	mmu-miR-8112<br>MIMAT0031418<br>chr6:71271371-71272101	CTCGAGacttcccctcaggaagtcagg<br>GAATTCggcgggaactcaccttctct	XhoI<br>EcoRI	205	0
No32	mmu-miR-211-3p<br>MIMAT0017059<br>chr7:64205506-64206211	GAATTCgtcctaagaatcagatcttggtgga<br>GGATCCacttcatttccagaagcagagaaga	EcoRI<br>BamHI	228	0
No33	mmu-miR-671-5p<br>MIMAT0003731<br>chr5:24591814-24592511	GAATTCctgcttctacaatgctgactacctg<br>GGATCCcgacaacggtggtacagttcct	EcoRI<br>BamHI	222	1
No34	mmu-miR-709<br>MIMAT0003499<br>chr8:84085799-84086486	GAATTCagggtagccttgaactcagagattt<br>GGATCCaaaacggactcctctaggttgttct	EcoRI<br>BamHI	278	1
No35	mmu-miR-1247-3p<br>MIMAT0014801	GAATTCctctcccactaggggtagctgtt<br>GGATCCctaaaaagcctcagcacacgttc	EcoRI<br>BamHI	264	1

	chr12:110277748-110278429				
No36	mmu-miR-1894-3p<br>MIMAT0007878<br>chr17:35917589-35918269	GAATTCcaatcgggtctcttaaatctcca<br>GGATCCtttctctgcccatctccttacc	EcoRI<br>BamHI	397	0
No37	mmu-miR-3154<br>MIMAT0035714<br>chr2:32317965-32318643	GAATTCcgggttggacactaggtaggagt<br>GGATCCgtagacggagtggaggtatcctctg	EcoRI<br>BamHI	287	0
No38	mmu-miR-5132-5p<br>MIMAT0020643<br>chrX:74023228-74023898	GAATTCgtgccgcttctacaaagtgatg<br>GGATCCgtgaggatgtgaacgaggtcag	EcoRI<br>BamHI	259	1
No39	mmu-miR-5627-5p<br>MIMAT0022385<br>chr12:44210013-44210673	GAATTCaagtgagtcgacgatttcctcatc<br>GGATCCctaagggtgagacctgagttttctg	EcoRI<br>BamHI	235	1
No40	mmu-miR-6236<br>MIMAT0024857<br>chr9:110280987-110281709	GAATTCccatacccttttcttttccactacct<br>GGATCCctgggaagacacccaacagtaactt	EcoRI<br>BamHI	289	0
No41	mmu-miR-6244<br>MIMAT0024864<br>chr9:52115323-52116039	GAATTCtaaaaatcttctctcggttgtcctg<br>GGATCCcatttctgattcccgactctatgat	EcoRI<br>BamHI	278	2
No42	mmu-miR-7005-5p<br>MIMAT0027914<br>chr2:180179455-180180123	GAATTCaccctggtgctcttctatgacttc<br>GGATCCatctccagcatgttatcctggtcta	EcoRI<br>BamHI	269	1
No43	mmu-miR-7653-5p<br>MIMAT0029812<br>chr11:78178527-78179187	GAATTCgaacggtgttcccagaactgtc<br>GGATCCatacaatggagtgggtgaaagagac	EcoRI<br>BamHI	243	0

Notes: Uppercase letters represent the restriction enzyme sites. Upper rows in each primer sequence represent the forward primers, while lower rows in the primer sequence represent the reverse primers.

Supplementary Table 2. Primer sequences used for the undertaking of quantitative PCR.

Gene name	Forward primer 5' → 3'	Reverse primer 5' → 3'
mouse ACTB	ggtgtgatggtgggaatgg	tcaggatacctctcttgctctgg
mouse FGF2	gcgacccacacgtcaaactac	gcacacactcccttgatagacac
human ACTB	gctcctcctgagcgcaag	catctgctggaaggtggaca
human FGF2	agcgaccctcacatcaagc	acggttagcacacactcctttg
human BMX	ctgaaaagtagcagcagcagtg	ccatgaaatctttgaggtggtg

Supplementary Table 3. Oligo sequences used for the construction of the 3'UTR plasmids for human GSK3B.

Plasmid's name	Oligo sequence 5' → 3'
3'UTR 1694 wild	AAACTAGCGGCCGCTAGTTCCTTCCCCACATGCCTTCT
	CTAGAAAAAGGAAAGGGAAAAATAGATGACTAGCGGCCGCTAGTTT
3'UTR 1694 mutated	AAACTAGCGGCCGCTAGTTCTTTCCCCACATACTTTCT
	CTAGAGAAAGTATGTGGGGAAAGAACTAGCGGCCGCTAGTTT
3'UTR 1774 wild	AAACTAGCGGCCGCTAGTTCACCCTCCTCATTGCCACCT
	CTAGAGGTGGCAATGAGGAGGGTGAACTAGCGGCCGCTAGTTT
3'UTR 1774 mutated	AAACTAGCGGCCGCTAGTTTACCCTCTTCATCGTCACCT
	CTAGAGGTGACGATGAAGAGGGTAAACTAGCGGCCGCTAGTTT
3'UTR 2899 wild	AAACTAGCGGCCGCTAGTTCCTTTATCCATGTCTCCT
	CTAGAGGAGACATGGATAAAGGAACTAGCGGCCGCTAGTTT
3'UTR 2899 mutated	AAACTAGCGGCCGCTAGTTCTTTTATCCACGTCCCCT
	CTAGAGGGGACGTGGATAAAAGAACTAGCGGCCGCTAGTTT
3'UTR 3248 wild	AAACTAGCGGCCGCTAGTCCTTCCATGCTCTCTCTCTCTT
	CTAGAAGAGAGAGAGAGCATGGAAGGACTAGCGGCCGCTAGTTT
3'UTR 3248 mutated	AAACTAGCGGCCGCTAGTCCTTTCATACTCCCTCTCCCTT
	CTAGAAGGGAGAGGGAGTATGAAAGGACTAGCGGCCGCTAGTTT
3'UTR 4864 wild	AAACTAGCGGCCGCTAGTACCCAACCCTCTTCCTTCT
	CTAGAGAAGGAAGAGGGTTGGGTACTAGCGGCCGCTAGTTT
3'UTR 4864 mutated	AAACTAGCGGCCGCTAGTACTCAACCTTTTTCTTTCT
	CTAGAGAAAGAAAAAGGTTGAGTACTAGCGGCCGCTAGTTT

Notes: Upper rows in each set of oligo sequences represent the forward oligo sequences, while lower rows in each set of oligo sequences represent the reverse oligo sequences.

Supplementary Table 4. Target sequence and oligo sequences used for the construction of the plasmids.

Name	Oligo sequence 5' → 3'
Scramble shRNA	GATCCgtgatactgttgtcagctgctgatcCTTCCTGTCAGAgatcagcagctgacaacagtatcacTTTTTG
	AATTCAAAAAgtgatactgttgtcagctgctgatcTCTGACAGGAAGgatcagcagctgacaacagtatcacG
GSK3B shRNA1	GATCCtcctgatactgctgtattaaaacCTTCCTGTCAGAgttttaatacagcagtatcaggaTTTTTG
	AATTCAAAAAtcctgatactgctgtattaaaacTCTGACAGGAAGgttttaatacagcagtatcaggaG
GSK3B shRNA2	GATCCcccaatgtttcgtatatctgttcCTTCCTGTCAGAgaacagatatacgaaacattgggTTTTTG
	AATTCAAAAAcccaatgtttcgtatatctgttcTCTGACAGGAAGgaacagatatacgaaacattgggG

Notes: Upper rows in each oligo sequence set represent the forward oligo sequences, while the lower rows in each oligo sequence set represent the reverse oligo sequences. Underlined solid lines highlight the sense sequences of the target genes, while underlined dot lines highlight the antisense sequence of the target genes.
